# COVID-19 Disease and SARS-CoV-2 Vaccination in Patients with Cancer

**DOI:** 10.33696/pharmacol.3.020

**Published:** 2021

**Authors:** Daniel A. King, Jeffrey Chi, Shreya Prasad Goyal, M. Wasif Saif

**Affiliations:** 1 Stanford University, Stanford, CA, USA; 2 Northwell Health Cancer Institute and Feinstein Institute of Research, Lake Success, NY, USA

**Keywords:** COVID-19, Cancer patients, Immunosup-pressed, Infection

Since the declaration of COVID-19 as a pandemic in March 2020 [1], there have been more than 100 million reported cases of COVID-19 worldwide and more than 2.1 million deaths [2]. The purpose of this editorial is to review recent updates regarding COVID-19 disease and SARS-CoV-2 vaccination in cancer patients.

Several studies have shown that cancer patients infected with COVID-19 disease have high rates of morbidity and mortality, and that a substantial portion of deaths in cancer patients are attributable to COVID-19 infection. As examples, a meta-analysis that pooled 52 studies of 18,650 cancer patients showed a case-fatality rate of 25.6% [3]. In a recent report comparing 41 and 35 cancer patients with and without COVID-19, respectively, the 30-day mortality of cancer patients with COVID-19 was 22%, compared to 0% in patients without COVID-19. A prospective cohort study estimated that greater than 90% of deaths in cancer patients with COVID-19 are attributable to COVID-19 [4]. Cancer patients are about twice as likely to contract infection with SARS-CoV-2 [5], the virus causing COVID-19.

Several factors underlie the risks of cancer patients to become infected with SARS-CoV-2 and their propensity toward developing COVID-19 complications. Patients with cancer have weakened immune systems leading to vulnerability to infection. In some cases, this is due to immune dysregulation intrinsic to the cancer, especially apparent in hematologic malignancies [4,6,7], but more broadly, immunosuppression is induced by cancer-directed therapy, by regimens that are cytotoxic to the immune system, either directly, such as with bone marrow transplantion [8], or indirectly, by cytotoxic chemotherapy [9]. Whereas patients affected by COVID-19 without cancer can clear SARS-CoV-2 viremia within 1–2 weeks, clearance is delayed for patients with solid tumors (29 days) [6], with hematologic malignancies (55 days) [6], and with hematologic malignancies undergoing stem-cell transplantation (longer than 2 months) [10]. Lymphopenia, which is predictive for severe COVID-19 disease, persists after SARS-CoV-2 infection, especially in patients with hematologic malignancies [6]. Separately, lung cancer is associated with severe COVID-19 disease and portends poorer outcome in the context of COVID-19, which is likely related to prior smoking history, leading to impaired pulmonary function and poor respiratory reserve [11]. Among patients with solid tumors, the diagnosis of cancer less than 5 years is associated with increased risk, especially in the first year, but the risk drops to baseline if the diagnosis is greater than 5 years [12]. Lastly, patients with cancer have a generally poorer substrate, with a constitution often already weakened by their cancer, and cancer patients are generally older, tend to be in overall poorer health and have comorbid conditions.

Vaccine development against SARS-CoV-2 manifested with extraordinary pace. The virus emerged as early as November 2019 [13,14] and the full viral sequence, a requisite for mRNA vaccine development [15], was published by January 2020 [16]. By the second half of 2020, at which point more than 120 candidate SARS-CoV2 vaccines were in development [17], mRNA vaccines showed excellent antibody responses without safety concerns in non-human primates [18], and in phase 1 studies [19,20], prompting their further developing into phase 3 trials. In December 2020, phase 3 COVID-19 trials were reported by Moderna [21] (mRNA-1273) and by a partnership between Pfizer and BioNTech [22] (BNT162b2). These trials had similar design, both randomizing participants to vaccine or placebo and compared the rate of symptomatic COVID-positive infections following completion of two-dose vaccination. In aggregate, these trials administered vaccines to more than 30 thousand participants; serious adverse events were rare and the vaccines were remarkably effective, with efficacies of 94.1% (Moderna [21]) and 94.6% (Pfizer [22]). Based on these randomized phase 3 data, the FDA issued emergency use authorization to both vaccines in December 2020, permitting administration to people at least 16 years (Pfizer-BioNTech) or 18 years (Moderna) of age. The mRNA vaccines require two doses and require freezer storage, factors which hinder distribution and full vaccination, especially in developing countries. Phase 3 trials are underway for at least 8 additional SARS-CoV-2 vaccines [23], including vaccines not requiring freezer storage. Interim analyses released in press releases by NovavaX of its two-dose spike-protein vaccine (NVX-CoV2373) [24], and by Johnson & Johnson, and its subsidary, Janssen, of its single- injection refrigerator-storage adenovirus viral vector vaccine (Ad.26.COV2.S) [25], are highly encouraging and may lead to future emergency use authorization. Similarly, the Chinese CoronaVac inactivated viral particle vaccine and the Russian Sputnik V adenovirus viral vector vaccine are of note because they were the first to be developed but trials demonstrating their effectiveness have also not yet undergone peer review as of January 2021 [23]. Distribution of COVID-19 vaccines is underway, with more than 70 million doses delivered among 57 countries as of late January 2021 [26].

The CDC developed a framework consisting of four ethical principles to guide distribution of vaccines [27], to ensure that allocation maximizes protection of those at risk of infection and at risk of morbidity or mortality to the virus [28]. Their guidelines call for staggered roll-out by phases, with highest priority, phase 1a, for health care personnel and long-term care facility residents, and phase 1b for frontline essential workers or patients at least 75 years old. Those not otherwise qualifying for the 75 million people among phase 1a or 1b, but who have cancer or other high-risk medical conditions, are included in phase 1c of vaccine distribution.

Data regarding safety and efficacy of COVID-19 vaccination in cancer patients is limited. Both phase 3 COVID-19 trials described above excluded patients with a known history of immunosuppressive therapy. The Pfizer study [22] explicitly excluded patients with a diagnosis of an immunocompromising condition. Only 3.7% of patients receiving the vaccine had a diagnosis of cancer, presumably not receiving active treatment, and while the adverse events eXperienced in this sub-group was not reported, the overall rate serious adverse events in the unselected study population was very low (<1%) [22]. The number of subjects with cancer was not reported on the Moderna trial. Taken together, limited empirical data exist for predicting safety in the cancer population. However, as mRNA vaccines do not deliver live virus, the National Center for Immunization and Respiratory Diseases advises that while the risks to immunocompromised patients are unknown, the vaccine is unlikely to pose a risk to safety [29]. Regarding efficacy, data suggest that the vaccination is likely to be effective even in patients undergoing cancer treatment. For example, it appears that most patients with solid malignancies affected by COVID-19, even with severe COVID-19 disease, are able to mount an immune response and produce antibodies against SARS-COV-2, although responses may be blunted in patients with hematologic malignancies [6].

For oncologists, the main question at hand is whether vaccination of patients undergoing active treatment for cancer merit vaccination for COVID-19. Emerging guidelines argue that the benefits generally outweigh the harms of vaccination. As nicely articulated by Dr. Steve Pergram, Co-Leader of the NCCN COVID-19 Vaccination Advisory Committee, “Cancer patients are not expected to be at risk for complications of the vaccine. They are at risk for acquisition and complications of the virus” [30].

The American Association of Cancer Research’s COVID-19 and Cancer Task Force recommend priority COVID-19 vaccination for cancer patients, especially those with hematologic malignancies, given an increased rate of severe COVID infection and death [31]. The National Comprehensive Cancer Network (NCCN), noting unknown vaccine efficacy in the setting of cancer care and a weakened immune system but acknowledging high-risk of COVID-19 associated complications, recommends that patients with cancer should be prioritized for vaccination and immunized when a vaccine is available to them (Table 1) [32]. The European Society for Medical Oncology (ESMO) recommends that immunocompromised patients with cancer have an increased risk of severe COVID-19 disease and should be vaccinated against the SARS-CoV-2 virus with high priority [33]. If possible, vaccination should be administered before the initiation of chemotherapy [34], but if it has already been initiated there is no clear guidance on timing around chemotherapy infusions [34,35]. It is possible that multiple doses (beyond the standard vaccination dosage guidelines) may be required in order to reach adequate seroconversion and seroprotective rates in cancer patients on chemotherapy [35].

Lessons from influenza vaccination provide an analogous vaccination model. Data suggest that cancer patients are able to mount an immune response to influenza [34,36], and on the basis of observational data, influenza vaccination is associated with lower mortality and absence of safety concerns [34,37]. On this basis, patients with active malignancies are recommended to receive the influenza vaccine [34], and may well be appropriate COVID-19 vaccine candidates.

Ultimately, cancer patients will need to be closely monitored after COVID-19 vaccination to assess for any adverse events, both related to COVID-19 (infection, severity and/or mortality) and cancer (complications, mortality) [33]. In time, further data are likely to additionally refine guidelines related to COVID-19 vaccination in the cancer population.

## Figures and Tables

**Table 1:** COVID-19 Vaccination Recommendations for Cancer Patients. Adapted from NCCN guidelines [32].

Patients Treatment/Cancer Type	Timing^†^
**Hematopoietic Cell Transplantation (HCT)/Cellular Therapy**	
Allogeneic Transplantation Autologous Transplantation Cellular therapy (e.g., CAR-T cell)	At least 3 months post- HCT/cellular therapy
**Hematologic malignancies**	
Receiving intensive cytotoxic chemotherapy (e.g. cytarabine/ anthracycline-based induction regimens for AML)	Delay until absolute neutrophil count (ANC) recovery
Marrow failure from disease and/or therapy expected to have limited or no recovery	When vaccine available
Long-term maintenance therapy (e.g., targeted agents for chronic lymphocytic leukemia or myeloproliferative neoplasms)	When vaccine available
**Solid tumor malignancies**	
Receiving cytotoxic chemotherapy	When vaccine available
Targeted therapy	When vaccine available
Checkpoint inhibitors and other immunotherapy	When vaccine available
Radiation	When vaccine available
Major surgery	Separate date of surgery from vaccination by at least a few days
**Caregivers and Household/Close Contacts (≥ 16 years of age)**	
Any time eligible to receive the vaccine	

† COVID-19 vaccines should be prioritized over other needed vaccines, as data on dual vaccination is not available to date. 14 days recommended between COVID-19 vaccines and other approved vaccines.
